# Antioxidant and Neuroprotective Effects of *N*-((3,4-Dihydro-2H-benzo[h]chromen-2-yl)methyl)-4-methoxyaniline in Primary Cultured Rat Cortical Cells: Involvement of ERK-CREB Signaling

**DOI:** 10.3390/molecules23030669

**Published:** 2018-03-15

**Authors:** Kyeongjun Lee, Chowee Park, Yeonsoo Oh, Heesoon Lee, Jungsook Cho

**Affiliations:** 1 College of Pharmacy, Dongguk University-Seoul, Goyang 10326, Korea; curian31@gmail.com (K.L.); we4638@naver.com (C.P.); dhdustn92@naver.com (Y.O.); 2 College of Pharmacy, Chungbuk National University, Cheongju 28160, Korea; medchem@chungbuk.ac.kr

**Keywords:** Alzheimer’s disease, chromene derivative, excitotoxicity, ERK1/2, CREB phosphorylation, antioxidant

## Abstract

Excitotoxicity and oxidative stress play vital roles in the development of neurodegenerative disorders including Alzheimer’s disease (AD). In the present study, we investigated the effect of *N*-((3,4-dihydro-2H-benzo[h]chromen-2-yl)methyl)-4-methoxyaniline (BL-M) on excitotoxic neuronal cell damage in primary cultured rat cortical cells, and compared to that of memantine, a non-competitive *N*-methyl-d-aspartate (NMDA) receptor antagonist clinically used to treat AD. We found that BL-M inhibited glutamate- or *N*-methyl-d-aspartate (NMDA)-induced excitotoxic cell damage. The IC_50_ value of BL-M against NMDA toxicity was comparable to that of memantine. BL-M potently inhibited intracellular reactive oxygen species generated by glutamate or NMDA. Additionally, it inhibited the formation of 1,1-diphenyl-2-picryl-hydrazyl radicals in vitro and lipid peroxidation in rat brain homogenates. In contrast, memantine showed minimal or negligible antioxidant activity. Western blotting and immunocytochemical analyses showed that BL-M, not memantine, increased the ERK1/2 phosphorylation and subsequent phosphorylation of cAMP response element-binding protein (CREB). The inhibition of NMDA toxicity by BL-M was dramatically reversed by U0126, a well-known MEK inhibitor, suggesting that ERK1/2-mediated CREB phosphorylation is required for the neuroprotective action. Collectively, in this study, we demonstrated the neuroprotective effect of a newly synthesized chromene derivative BL-M and its underlying action mechanism(s). In contrast to memantine, BL-M exhibited marked antioxidant activity. Furthermore, it enhanced the ERK-mediated phosphorylation of CREB, which plays a crucial neuroprotective role. Our findings suggest that BL-M may be beneficial for AD and other neurodegenerative disorders associated with excitotoxicity as well as oxidative stress.

## 1. Introduction

Glutamate (Glu) is a major excitatory amino acid neurotransmitter that plays a crucial role in both physiological and pathological conditions of the central nervous system (CNS). It is involved in most aspects of normal brain function including cognition, memory, and learning [1]. At the same time, under certain pathological conditions, it acts as an excitotoxin via overactivation of Glu receptors [2]. Glu binds to and activates specific ionotropic and metabotropic receptors throughout the CNS that have diverse effects on neural excitability [3]. It is well recognized that excessive amounts of Glu in the brain result in excitotoxic cell death via overstimulation of Glu receptors. Overstimulation of *N*-methyl-d-aspartate (NMDA) receptor, one of the ionotropic Glu receptors, has been extensively studied to elucidate its role in many brain diseases including Alzheimer’s disease (AD) [4,5].

AD is the most common cause of dementia and a chronic neurodegenerative disease characterized by symptoms of cognitive impairment and memory loss [6]. Senile plaques, mostly insoluble extracellular deposits of amyloid beta (A_β_), and neurofibrillary tangles, intracellular aggregates of hyperphosphorylated tau protein, are the primary characteristic features of AD. Besides, several pathophysiological factors including cholinergic deficiency, oxidative stress, mitochondrial dysfunction and chronic inflammation have been proposed in AD [7,8]. Despite efforts to develop novel disease-modifying drugs, AD patients still have a very narrow range of treatment options available.

Acetylcholinesterase inhibitors (AChEIs) and memantine are the currently available drugs used to treat AD in clinical situations. AChEIs such as donepezil, rivastigmine, and galantamine are believed to enhance cholinergic neurotransmission through reversible inhibition of the enzyme that catalyzes the breakdown of Ach [8]. On the other hand, the principal action mechanism of memantine, a non-competitive NMDA antagonist, involves blockade of current flow through NMDA receptor-ion channel [9]. Activation of the NMDA receptor is followed by Ca^2+^ ion influx into the post-synaptic neurons. Under physiological conditions, activation of NMDA receptor triggers a signaling cascade that regulates synaptic plasticity such as long-term potentiation underlying learning and memory [10,11]. In case of AD, however, NMDA receptors are continuously over-activated, causing high levels of Ca^2+^ influx [12]. Prolonged Ca^2+^ overload in the post-synaptic neurons generates excitotoxicity, resulting in a gradual neurodegenerative effect [11,12].

**Figure 1:**
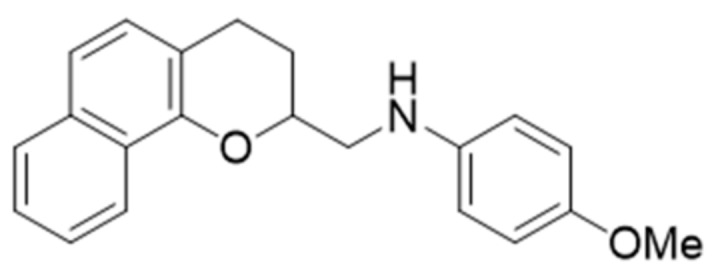
The chemical structure of *N*-((3,4-dihydro-2H-benzo[h]chromene-2-yl)methyl)-4-methoxyaniline (BL-M).

A novel chromene derivative, *N*-((3,4-dihydro-2H-benzo[h]chromene-2-yl)methyl)-4-methoxyaniline (BL-M, Figure 1), was originally synthesized as a potential nuclear factor kappa B (NF-κB) inhibitor that plays a key role in regulating inflammation [13]. Among the 25 compounds of 3,4-dihydro-2H-benzo[h]chromene derivatives, several compounds showed potent inhibition of LPS-induced NF-κB transcriptional activity in RAW 264.7 macrophages, while others including BL-M showed only mild to moderate inhibition of NF-κB [13]. Subsequently, BL-M was reported to exhibit anti-oxidative and anti-inflammatory activities in BV2 microglial cells through inhibition of nuclear translocation of NF-κB [14]. However, the pharmacological actions of BL-M in neurons have yet to be investigated.

In the present study, we evaluated the protective effect of BL-M on excessive excitatory insults induced by Glu or NMDA in primary cultured rat cortical neurons. Since BL-M exhibited marked inhibition of Glu- or NMDA-induced neuronal cell death, we compared its effect to that of memantine. In addition, the antioxidant properties of BL-M and signaling molecule(s) involved in the neuroprotective action were investigated.

## 2. Results

### 2.1. Effects of BL-M and Memantine on Glu- or NMDA-Induced Excitotoxicity in Primary Cultured Rat Cortical Cells

**Figure 2:**
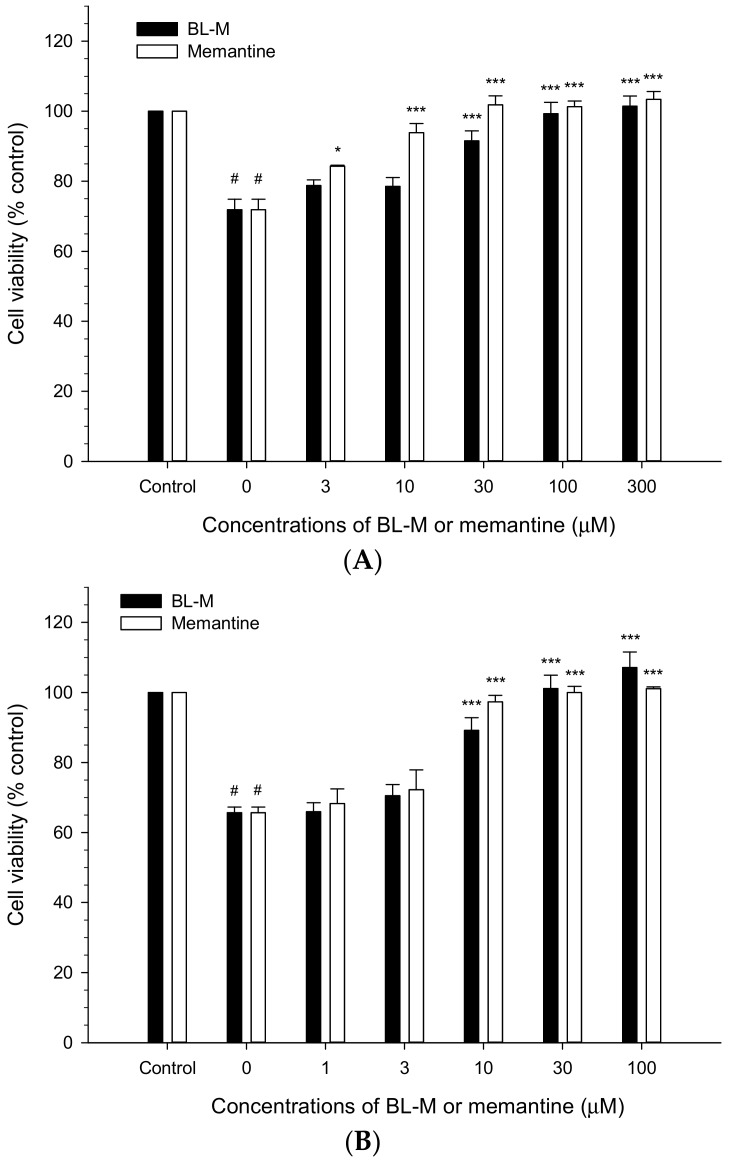
Neuroprotective effects of BL-M and memantine on Glu- or *N*-methyl-d-aspartate (NMDA)-induced excitotoxic neuronal cell death in the primary cultured rat cortical cells. The cultured cells (10–11 days in vitro) were exposed to 100 μM Glu (**A**) or 300 μM NMDA (**B**) for 15 min in the absence or presence of the indicated concentrations of BL-M or memantine, as described in the Section 4. Cell viability was assessed using the MTT reduction assays followed by 22–24 h incubation at 37 °C after the exposure, and expressed as % of control-treated cells (# *p* < 0.001 vs. control; * *p* < 0.05, *** *p* < 0.001 vs. Glu- or NMDA-treated cells, respectively). Each data represents the mean ± S.E.M. from at least three independent experiments.

The neuroprotective effect of BL-M was evaluated against the excitotoxic neuronal cell death induced in primary cultured rat cortical cells and compared to that of memantine. Consistent with our previous reports, the viability of the cultured cells exposed to 100 μM Glu was significantly reduced to approximately 70% compared to the control cells. Co-treatment with BL-M or memantine decreased the Glu-induced excitotoxicity in a concentration-dependent manner (Figure 2A). The neuroprotective effect of BL-M was statistically significant at 30 μM and above. Memantine also significantly restored the cell viability at 3 μM and above. The calculated IC_50_ values, the concentrations exhibiting 50% inhibition, of BL-M and memantine against the Glu-induced excitotoxic cell death were 16.95 and 3.32 μM, respectively. Based on their IC_50_ values, memantine was approximately 5-fold more potent than BL-M. Similarly, exposure of cells to 300 μM NMDA decreased the cell viability to approximately 65% of control (Figure 2B). BL-M and memantine restored the cell viability in concentration-dependent manners. The respective IC_50_ values of BL-M and memantine against the NMDA-induced toxicity were 6.92 and 4.68 μM, showing comparable potency.

### 2.2. Effects of BL-M and Memantine on Glu- or NMDA-Induced ROS Generation in Primary Cultured Rat Cortical Cells

**Figure 3:**
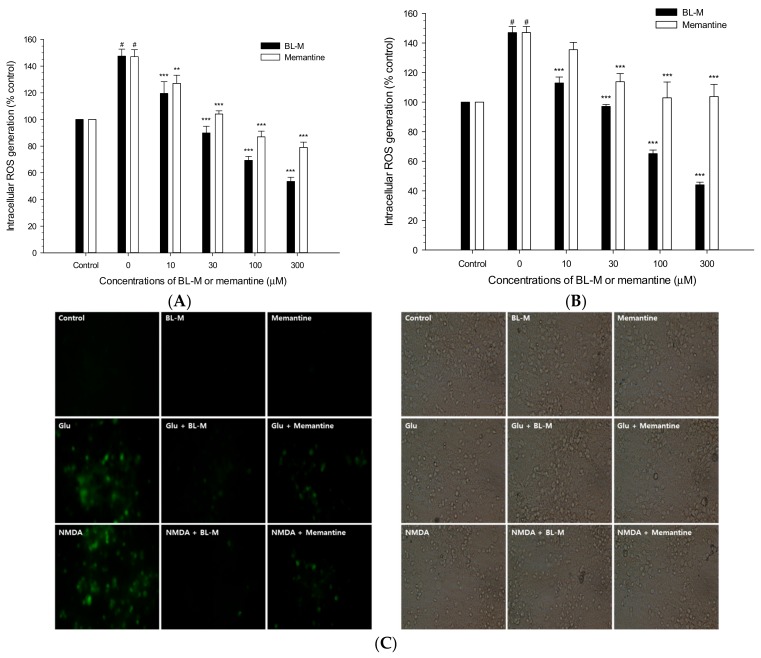
Inhibition by BL-M or memantine of Glu- or NMDA-induced intracellular ROS generation in primary cultured rat cortical cells. The cultured cells (10–11 days *in vitro*) were incubated with 10 μM DCFH-DA for 30 min and exposed to 100 μM Glu (**A**) or 300 μM NMDA (**B**) in the absence or presence of the indicated concentrations of BL-M or memantine, as described in the Section 4. The levels of intracellular ROS were measured at 2 h after the exposure to the respective insults, and expressed as % of control-treated cells. (# *p* < 0.001 vs. control; ** *p* < 0.01, *** *p* < 0.001 vs. Glu- or NMDA-treated cells, respectively). Each data represents the mean ± S.E.M. from at least three independent experiments. Representative epifluorescent (left) and bright-field (right) images of cells exposed to Glu or NMDA with or without BL-M or memantine at 10 μM are shown (**C**).

To examine whether BL-M inhibited intracellular ROS generation induced by Glu or NMDA, the levels of ROS were measured using DCFH-DA as a fluorescent probe. The ROS level was increased up to approximately 150% of control by Glu treatment, and the increased ROS was effectively inhibited by BL-M (Figure 3A). Similarly, the increased level of intracellular ROS by NMDA treatment was markedly reduced by BL-M (Figure 3B). Memantine also decreased Glu- or NMDA-induced ROS generation. However, it exhibited less potent inhibition of ROS than BL-M (Figure 3A,B). The strong inhibition of ROS generation by BL-M at the concentration of 10 μM was further confirmed by epifluorescence study (Figure 3C).

### 2.3. Effects of BL-M and Memantine on Lipid Peroxidation and DPPH Radicals

**Figure 4:**
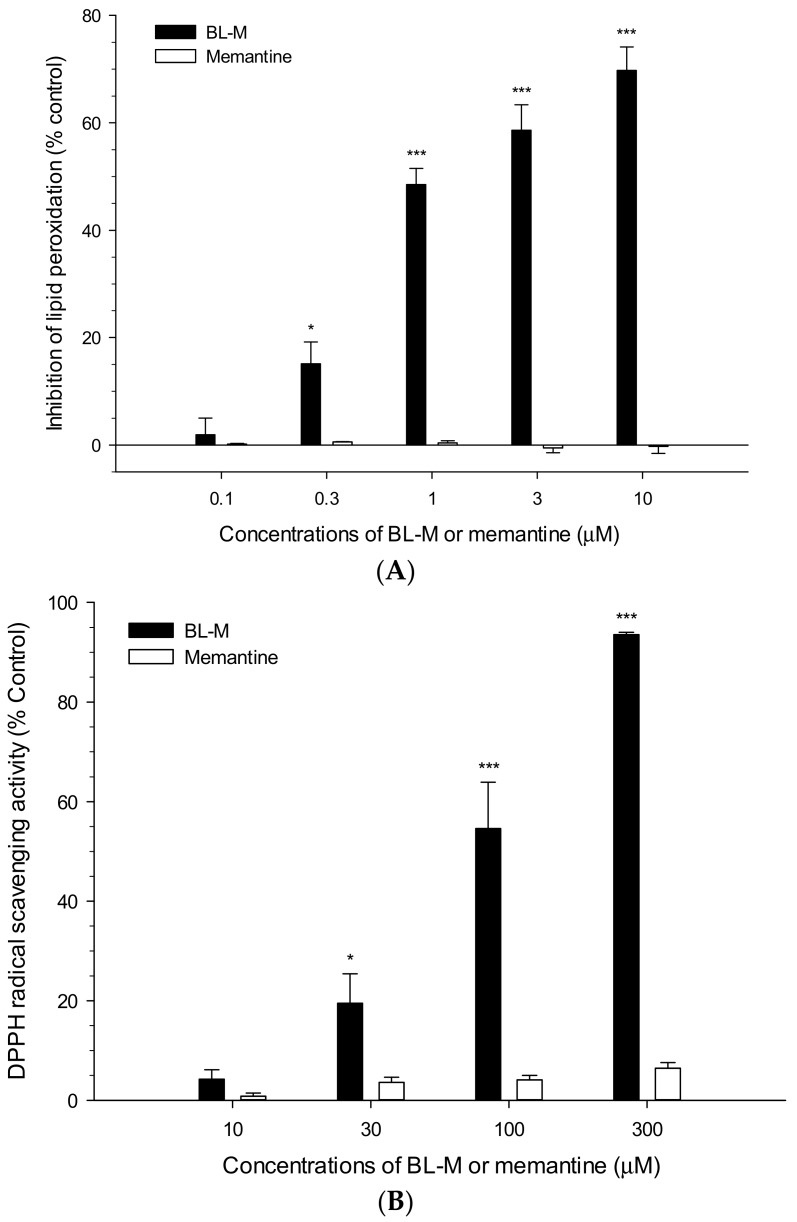
Differential antioxidant activities of BL-M and memantine. Lipid peroxidation induced by Fe^2+^ and l-ascorbic acid in rat brain homogenates (**A**) and DPPH radical scavenging activities (**B**) were assessed in the absence or presence of BL-M or memantine, as described in the Section 4 (* *p* < 0.05, *** *p* < 0.001 vs. control). Each data represents the mean ± S.E.M. from at least three independent experiments.

We then compared the antioxidant property of BL-M to that of memantine using in vitro assays of lipid peroxidation and DPPH radical scavenging activity. Consistent with our previous report [14], BL-M strikingly inhibited lipid peroxidation initiated by Fe^2+^ and l-ascorbic acid in rat brain homogenates (Figure 4A). However, memantine showed no effect on lipid peroxidation under our experimental conditions at the concentration ranges of 0.1~10 μM (Figure 4A) or even at higher concentrations (up to 100 μM, data not shown). Similarly, BL-M noticeably eliminated DPPH free radicals as reported previously [14], whereas memantine had a minimal or negligible effect on the stable free radicals produced by DPPH at the concentrations tested in this study (Figure 4B). These results suggest that, although both BL-M and memantine exhibited comparable neuroprotective effects, their underlying action mechanism(s) may be distinct.

### 2.4. Effects of BL-M and Memantine on NMDA-Induced Nuclear Translocation of NF-κB in Primary Cultured Rat Cortical Cells

**Figure 5:**
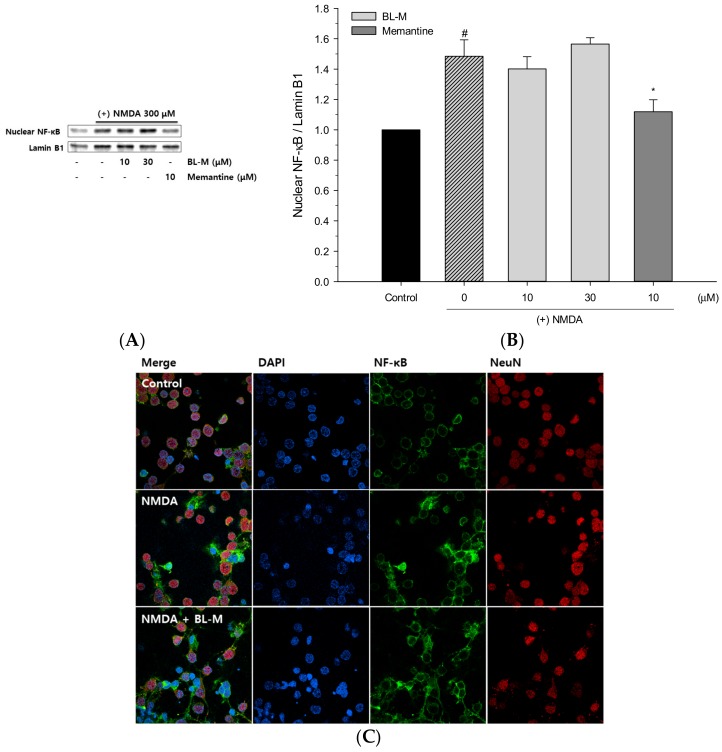
Effects of BL-M and memantine on NMDA-induced nuclear translocation of NF-κB in primary cultured rat cortical cells. Nuclear fractions obtained from the cells treated with NMDA in the absence or presence of BL-M or memantine at the indicated concentrations were analyzed by Western blotting using a primary antibody of anti-NF-κB p65 (**A**). Lamin B1 was used for loading control. The quantitative analysis was expressed as relative changes with respect to the control group (**B**) (# *p* < 0.001 vs. control; * *p* < 0.05 vs. NMDA-treated group). Each data represents the mean ± S.E.M. from three independent experiments. Representative images of immunocytochemical staining conducted in the cells exposed to NMDA with or without BL-M at 10 μM are shown (**C**).

BL-M was originally designed as one of the series of potential NF-κB inhibitors, and previously reported to moderately inhibit LPS-induced NF-κB transcriptional activity in RAW 264.7 macrophages and in BV-2 microglial cells [13,14]. Therefore, we first tested its effect on NF-κB translocation induced by NMDA in our culture. When the cells were treated with NMDA, the level of p65 NF-κB in the nuclear fraction was increased (Figure 5). Interestingly, however, BL-M did not inhibit the nuclear NF-κB level at the concentrations tested in this study (Figure 5A,B). In agreement with these findings, the immunocytochemical analyses of cells exposed to NMDA and BL-M showed that the NMDA-induced nuclear NF-κB was not dramatically inhibited by BL-M at the concentration of 10 μM (Figure 5C). In contrast, memantine significantly suppressed the NMDA-induced NF-κB translocation (Figure 5A,B).

### 2.5. Effect of BL-M on the Phosphorylation of cAMP Response Element-Binding (CREB) Protein in Primary Cultured Rat Cortical Cells

**Figure 6:**
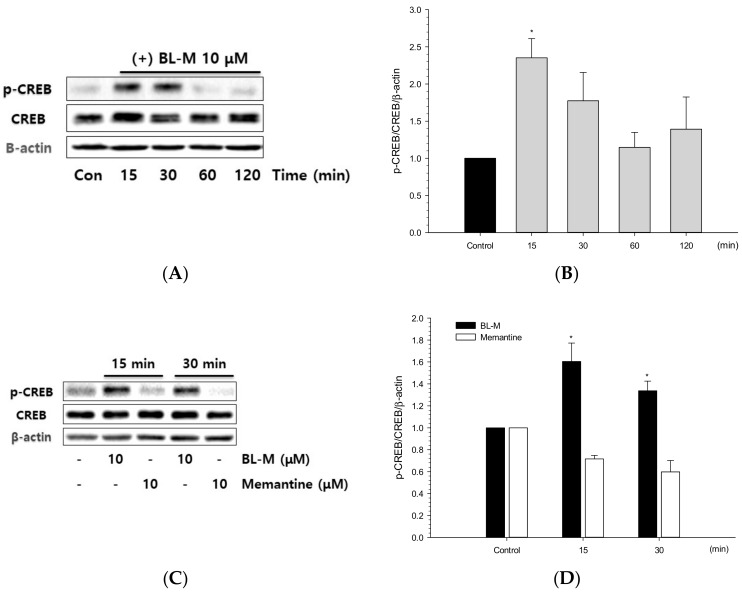
Phosphorylation of CREB by BL-M in primary cultured rat cortical cells. Cells (10–11 days in vitro) were treated with either BL-M alone (**A**) or BL-M or memantine (**C**) for the indicated periods of time, and the cell lysates were analyzed by Western blotting with a primary antibody of anti-phospho-CREB (Ser133). Total CREB and β-actin were used for loading control. The quantitative analyses were expressed as relative changes with respect to the respective control group (**B**, **D**) (* *p* < 0.05 vs. control). Each data represents the mean ± S.E.M. from at least three independent experiments.

CREB is a transcription factor that is known to play a crucial role in neuronal cell survival. In an attempt to identify the signaling molecule(s) mediating the neuroprotective effect of BL-M, we investigated its role in CREB phosphorylation. As illustrated in Figure 6A and B, BL-M treatment of the cultured cells substantially increased phosphorylation of CREB, with the maximal effect at 15 min. The CREB phosphorylation gradually declined to the control level over 2 h. In contrast, memantine did not induce the phosphorylation of CREB at 15 or 30 min of treatment (Figure 6C,D).

### 2.6. Effect of BL-M on the ERK1/2-CREB Signaling in Primary Cultured Rat Cortical Cells

**Figure 7:**
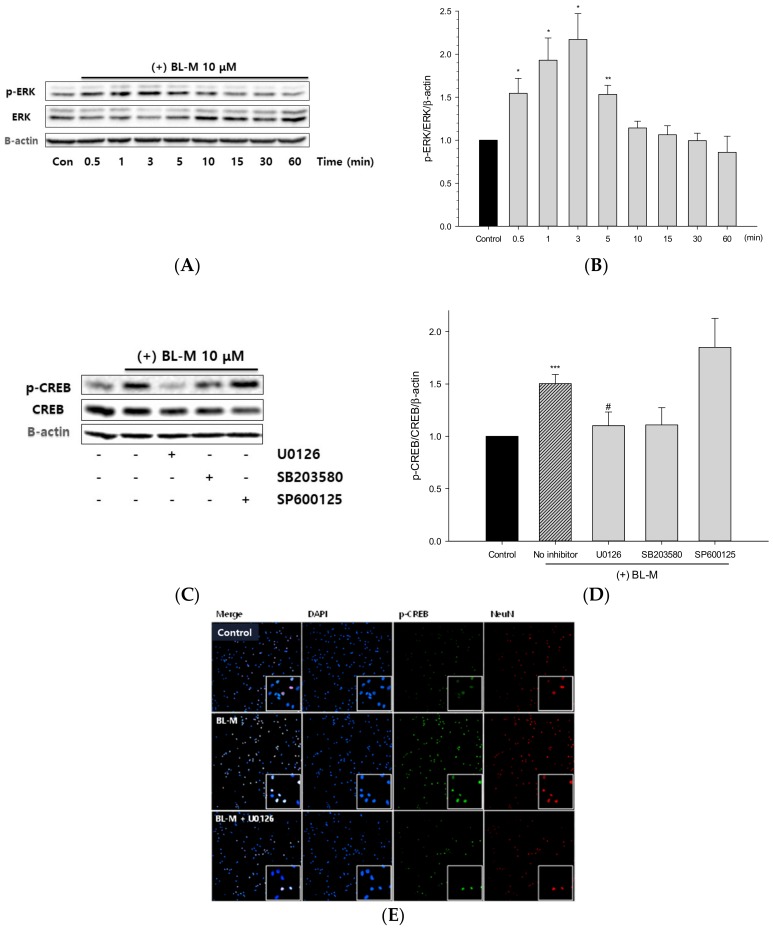
ERK1/2-mediated CREB phosphorylation by BL-M in primary cultured rat cortical cells. Cells (10–11 days in vitro) were treated with BL-M for the indicated periods of time, and the cell lysates were analyzed by Western blotting using phospho-ERK1/2 antibody, as described in the Section 4 (**A**). Total ERK1/2 and β-actin were used for loading control. The quantitative analysis was expressed as relative changes with respect to the control group (**B**). The levels of CREB phosphorylation in the cells pretreated with kinase inhibitors were analyzed by Western blotting with a primary antibody of anti-phospho-CREB (Ser133) (**C**), and quantitatively expressed as relative changes with respect to the control group (**D**). Each data represents the mean ± S.E.M. from at least three independent experiments. Representative images of immunocytochemical staining conducted in the cells pretreated with U0126 using phospho-CREB antibody are shown (**E**) (* *p* < 0.05, ** *p* < 0.01, *** *p* < 0.001 vs. control; # *p* < 0.05 vs. BL-M treated group).

To elucidate the upstream kinase involved in the CREB phosphorylation, we assessed BL-M-induced phosphorylation of ERK1/2. As shown in Figure 7A and B, BL-M induced time-dependent phosphorylation of ERK, with the maximal effect at 3 min.

In order to examine whether ERK1/2 phosphorylates CREB, cells were pretreated for 1 h with U0126, a well-known MEK inhibitor, followed by treatment with BL-M. Based on our Western blotting analysis, the BL-M-induced CREB phosphorylation was strongly inhibited by U0126 (Figure 7C,D). To exclude the possible involvement of p38 MAP kinase and JNK, we additionally tested the effects of the respective kinase inhibitors on CREB phosphorylation. Unlike U0126, the p38 MAP kinase inhibitor SB203580 and JNK inhibitor SP600125 did not induce significant inhibition of CREB phosphorylation (Figure 7C,D). Using immunocytochemical fluorescence staining, we confirmed that U0126 treatment reduced the BL-M-induced CREB phosphorylation (Figure 7E). These results indicate that ERK1/2, not p38 MAP kinase or JNK, is involved in the BL-M-induced phosphorylation of CREB in the cultured cortical cells.

### 2.7. Effects of Kinase Inhibitors on the Neuroprotective Effect of BL-M in Primary Cultured Rat Cortical Cells

**Figure 8:**
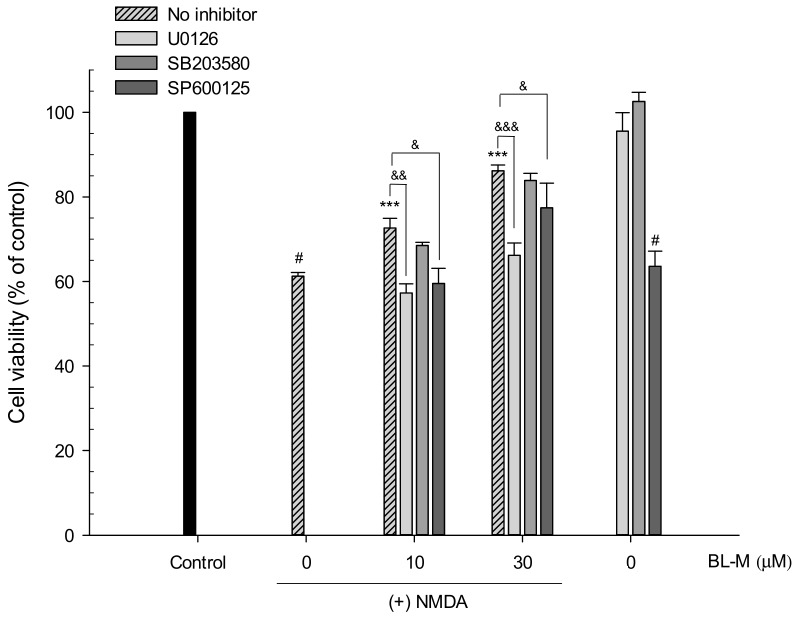
Reversal of the neuroprotective effect of BL-M by U0126 in primary cultured rat cortical cells. Cells (10–11 days in vitro) were pretreated with kinase inhibitors and exposed to 300 μM NMDA for 15 min in the absence or presence of the indicated concentrations of BL-M. Cell viability was assessed by MTT reduction assays after 22–24 h of incubation at 37 °C, as described in the Section 4. (# *p* < 0.001 vs. control; *** *p* < 0.001 vs. NMDA-treated cells; & *p* < 0.05, && *p* < 0.01, &&& *p* < 0.001 vs. NMDA + BL-M-treated cells). Each data represents the mean ± S.E.M. from at least three independent experiments.

We finally tested whether the BL-M-induced phosphorylations of ERK1/2 and CREB were required for its neuroprotective action. As shown in Figure 2B, BL-M exhibited neuroprotective effects at both 10 and 30 μM concentrations, restoring the cell viability decreased by NMDA treatment (Figure 8). The neuroprotective effect of BL-M was almost completely inhibited by U0126. In contrast, SB203580 failed to inhibit the neuroprotective role of BL-M. Interestingly, SP600125 appeared to significantly decrease the viability of cells treated with BL-M and NMDA. However, the inhibitor itself exhibited intensive toxicity at the concentration used in this study (Figure 8). These findings suggest that the phosphorylation of ERK1/2, as well as subsequent phosphorylation of CREB by ERK1/2, plays an essential role in the neuroprotective action of BL-M.

## 3. Discussion

BL-M is a novel chromene derivative originally synthesized as a potential NF-κB inhibitor [13]. A previous study using BV-2 microglial cells reported anti-oxidative and anti-inflammatory activities of BL-M [14]. In the present study, we investigated the neuroprotective effect of BL-M on excitotoxicity using primary cultured rat cortical cells and compared to that of memantine. BL-M was found to inhibit Glu- or NMDA-induced excitotoxic neuronal cell death. Memantine, a well-known NMDA receptor antagonist, also inhibited the Glu- or NMDA-induced excitotoxicity in our culture. Although both BL-M and memantine showed comparable neuroprotection against the NMDA-induced toxicity, only BL-M exhibited strong antioxidant activities, inhibiting intracellular ROS generation, lipid peroxidation, and DPPH radicals. We further elucidated signaling molecule(s) mediating the neuroprotective effect of BL-M. We found that the phosphorylation of ERK1/2-CREB pathway was required for the neuroprotective effect of BL-M. Collectively, we demonstrate in this study the neuroprotective effect of a newly synthesized chromene derivative BL-M and its underlying mechanisms of action. In contrast to memantine, BL-M exerts neuroprotective effect via distinct antioxidant activity and ERK-CREB signaling. Our findings suggest that BL-M may be beneficial for AD and other neurodegenerative disorders associated with excitotoxicity as well as oxidative stress.

Glu receptors including NMDA receptor play crucial roles in learning and memory as regulators of neural plasticity [1]. However, excessive stimulation of these receptors results in neurotoxicity [15]. The Glu-induced neurotoxicity, also called excitotoxicity, is one of the key mechanisms of neuronal injury in neurodegenerative diseases such as AD [4,5]. To determine the effect of BL-M on the excitotoxicity, we treated primary cultured rat cortical cells with Glu or NMDA. As reported previously, treatment of our cultured cells with Glu or NMDA caused marked reduction of cell viability to 60–70% when compared to the control cells (Figure 2). BL-M effectively inhibited the Glu- or NMDA-induced excitotoxicity (Figure 2). The calculated IC_50_ values revealed that BL-M inhibited NMDA-induced toxicity more potently than Glu toxicity, implying some degree of selectivity for NMDA receptor. The neuroprotective effect of BL-M was compared to that of memantine, a well-known NMDA antagonist, tested in this study as a reference. Memantine also inhibited the Glu- or NMDA-induced excitotoxicity. The IC_50_ value of memantine against NMDA toxicity was 4.68 μM, which was observed at the same concentration ranges reported previously [16]. Whereas BL-M exhibited nearly 5-fold less potent inhibition of Glu toxicity than memantine, it inhibited NMDA toxicity with almost comparable potency of memantine.

ROS and free radicals have been regarded as important therapeutic targets in many types of neurodegenerative disorders including AD [17]. The brain of AD patients shows a significant oxidative damage associated with A_β_ plaques and neurofibrillary tangles [18]. Moreover, lipid peroxidation has been considered as a major biomarker of mild cognitive impairment associated with AD [19]. It is believed to be induced not only by A_β_, neurofibrillary tangles, or even aging but also by excessive excitatory neurotransmitter stimulation [20]. Therefore, attempts to reduce free radicals and oxidative stress represent important therapeutic strategies in AD.

As reported earlier [21] and shown in this study (Figure 3), treatment of the cultured cells with Glu or NMDA significantly augmented the intracellular ROS levels. The Glu- or NMDA-induced ROS were dramatically inhibited by BL-M (Figure 3). Although memantine also inhibited intracellular ROS production, it exhibited less potent inhibition than BL-M. Similarly, BL-M showed marked antioxidant activities, inhibiting lipid peroxidation in rat brain homogenates as well as scavenging DPPH radicals (Figure 4). However, memantine exhibited only minimal or negligible in vitro antioxidant activity (Figure 4). These findings suggest that, although both BL-M and memantine exhibited neuroprotection against excitotoxic cell death, the mechanisms underlying their neuroprotective effects may be different. BL-M, not memantine, was shown to exhibit marked antioxidant activity in both cultured cells and in in vitro cell-free bioassays. The antioxidant property of BL-M may contribute to its neuroprotective action.

To elucidate molecular mechanism(s) or signaling molecule(s) mediating the neuroprotective effect of BL-M, we first attempted to examine if BL-M inhibits the NMDA-induced NF-κB translocation. In the nervous system, NF-κB is known to be involved in synaptic processes and neurotransmission. In addition, inducible NF-κB plays an important role in inflammation and neural stem cell proliferation in the CNS [22]. BL-M was originally synthesized as a derivative of KL-1156 (6-hydroxy-7-methoxychroman-2-carboxylic acid phenyl amide), which was reported as a prominent inhibitor of NF-κB translocation [13]. BL-M was reported to inhibit NF-κB transcriptional activity in RAW 264.7 macrophages, with the IC_50_ value of 44.5 μM [13]. Subsequently, BL-M was shown to suppress inflammatory mediators by inhibiting LPS-induced translocation of NF-κB in BV-2 microglial cells [14]. Interestingly, however, in the cultured rat cortical cells, NMDA-induced nuclear translocation of NF-κB was not inhibited by BL-M at the concentrations tested in this study (Figure 5A–C). Further study is required to elucidate whether the inhibition of NF-κB by BL-M is cell type-specific. It may also be possible that higher concentration of BL-M than 30 μM is required to exhibit inhibition of NMDA-induced NF-κB translocation in neurons. Memantine, in contrast, inhibited the NMDA-induced NF-κB translocation at 10 μM (Figure 5), providing another evidence of different mechanisms associated with the neuroprotective actions of BL-M and memantine.

Among the many transcription factors, CREB is known to play crucial roles in the development of the nervous system and in neuroprotection [23,24]. CREB proteins are activated via phosphorylation by various kinases including MAP kinase, and bind to DNA sequences and regulate the downstream genes [25,26]. We found a time-dependent phosphorylation of CREB by BL-M (Figure 6A,B). In contrast, memantine did not induce CREB phosphorylation (Figure 6C,D). To determine the upstream kinase, ERK1/2 phosphorylation was investigated in the BL-M-treated cells. As shown in Figure 7A and B, BL-M enhanced ERK phosphorylation in a time-dependent fashion. To confirm whether ERK1/2 is the upstream kinase of CREB, the effect of a selective MEK inhibitor U0126 was evaluated. The increased CREB phosphorylation by BL-M was markedly inhibited by U0126 (Figure 7C–E), indicating that ERK1/2 was the upstream kinase of CREB. Any possible involvement of other members of the MAP kinase family, p38MAP kinase and JNK, in CREB phosphorylation was excluded since their respective inhibitors SB203580 and SP600125 did not show significant changes in CREB phosphorylation (Figure 7C,D). These results indicate that BL-M enhanced phosphorylation of CREB via ERK1/2 pathway.

Finally, we examined whether the BL-M-induced activation of ERK-CREB pathway plays a crucial role in its neuroprotective action. As illustrated in Figure 8, the neuroprotective effect of BL-M was almost completely inhibited by U0126, not by SB203580. Although SP600125 appeared to significantly decrease the neuroprotective effect of BL-M, it may be due to the toxicity of the inhibitor itself (Figure 8). Moreover, as showed earlier (Figure 7C,D), CREB phosphorylation was not inhibited by SP600125. These results indicate that phosphorylation of ERK1/2, as well as subsequent phosphorylation of CREB, plays an essential role in the neuroprotective action of BL-M.

Several studies suggest that phosphorylation of CREB is associated with the expression of brain-derived neurotrophic factor (BDNF), which is a member of the neurotrophin family of growth factors [27,28]. Further studies are in progress to elucidate the involvement of BDNF in the neuroprotective action by BL-M.

Collectively, we demonstrate in this study the neuroprotective effect of a newly synthesized chromene derivative BL-M and its underlying mechanisms of action. Although both BL-M and memantine exhibited comparable neuroprotection against NMDA-induced excitotoxic neuronal cell death, the underlying mechanisms were distinct. Unlike memantine, BL-M exhibited marked antioxidant activity, which may contribute, at least in part, to its neuroprotective action. Surprisingly, inhibition of NMDA-induced NF-κB translocation may not be involved in the neuroprotective effect of BL-M. Instead, BL-M-induced phosphorylation of ERK1/2 and CREB plays a crucial role in the neuroprotective action. Based on our findings, BL-M may be beneficial for AD and other neurodegenerative disorders associated with excitotoxicity as well as oxidative stress.

## 4. Materials and Methods

### 4.1. Materials

Fetal bovine serum (FBS), horse serum (HS), minimum essential medium (MEM), and antibiotic-antimycotic solution were purchased from Gibco BRL (Grand Island, NY, USA). Laminin, cytosine arabinoside, L-Glu, 3-(4,5-dimethylthiazol-2-yl)-2,5-diphenyltetrazolium bromide (MTT), 2′,7′-dichlorofluorescin diacetate (DCFH-DA), anti-β-actin, and SP600125 were procured from Sigma-Aldrich (St. Louis, MO, USA). NMDA was obtained from Tocris Bioscience (Bristol, UK). Anti-phospho-CREB (Ser133), anti-phospho-p44/42 MAPK (Thr202/Tyr204), NF-κB p65, U0126, horseradish peroxidase (HRP)-conjugated anti-rabbit immunoglobulin G (IgG) and anti-mouse IgG were purchased from Cell Signaling Technology (Danvers, MA, USA). Anti-lamin B1 and SB203580 were purchased from Abcam (Cambridge, MA, USA).

### 4.2. Preparation of N-((3,4-Dihydro-2H-benzo[h]chromene-2-yl)methyl)-4-methoxyaniline (BL-M)

BL-M (Figure 1) was synthesized from 1-(1-hydroxynaphthalen-2-yl) ethanone via a series of reactions as previously described [13,14]. The final compound was purified by flash column chromatography (ethyl acetate/hexanes). Thin layer chromatography, performed on E Merck silica gel GF-254 pre-coated plates, showed only a single spot under UV illumination. IR, ^1^H-NMR, ^13^C-NMR, and mass spectra were performed to further identify the synthesized compound (see Supplementary Materials in ref. [14] in detail). For experiments, a 100 mM stock solution was prepared in dimethyl sulfoxide (DMSO) as a solvent and diluted to 10 mM before use.

### 4.3. Animals

Timed-pregnant Sprague-Dawley (SD) rats were purchased from Daehan Biolink (Chungbuk, Korea). The animals were housed in an animal room with a controlled temperature (22 ± 2 °C) and a 12 h dark-light cycle. All animals were allowed a standard chow diet and water *ad libitum*. Whole experimental steps were carried out according to the international guidelines (Guide for the Care and Use of Laboratory Animals, Institute of Laboratory Animal Resources, Commission on Life Sciences, National Research Council, USA; National Academy Press: Washington, D.C., 1996). The rationale, design, and protocols of the animal experiments were approved by the Institutional Animal Ethical Committee of Dongguk University prior to the study (Approval No. IACUC-2013-005 and IACUC-2016-035-2).

### 4.4. Primary Culture of Rat Cortical Cells

Mixed rat cerebrocortical cells containing neuronal and non-neuronal cells were separated from the brains of SD rat embryos at 17-days of gestation as described previously [29,30,31]. Briefly, cerebral cortices of the embryos were dissected and dissociated mechanically into single cells by trituration using fire-polished Pasteur pipettes. The isolated cells were plated in MEM supplemented with 5% FBS, 5% HS and 1% antibiotic-antimycotic agent at a density of 6 × 10^5^ cells/well of a 24-well culture plate (BD Falcon, Franklin Lakes, NJ, USA) or at a density of 6 × 10^6^ cells per 35 mm culture dish (Corning, NY, USA) pre-coated with laminin and poly-l-lysine. For immunocytochemical staining experiments, cells were plated at a density of 3 × 10^5^ cells per microscope cover glass (Marienfeld GmbH, Lauda-Königshofen, Germany), placed on each well of a 24-well culture plate. The cells were incubated at 37 °C in a humidified atmosphere of 95% air/5% CO_2_. At 7 days after plating, proliferation of non-neuronal cells was arrested by the addition of 10 μM cytosine arabinoside. All experiments were conducted at 10–12 days after plating.

### 4.5. Treatment of Cultured Cells

Cells were washed and maintained in serum-free media. After 2 h of serum starvation, cells were treated with different concentrations of BL-M or memantine. In order to evaluate the excitotoxic neuronal damage induced by excitotoxic insults, cultured cells were exposed to 100 μM Glu in *N*-(2-hydroxyethyl)piperazine-*N′*-(2-ethanesulfonic acid) (HEPES)-buffered control salt solution (HCSS, 120 mM NaCl, 5.4 mM KCl, 1.6 mM MgCl_2_∙6H_2_O, 2.3 mM CaCl_2_∙2H_2_O, 15 mM glucose, 10 mM NaOH, 20 mM HEPES, pH 7.4) or 300 μM NMDA in Mg^2+^-free HCSS for 15 min. Following the treatment, cells were washed and incubated in serum-free media for 22–24 h in the incubator. To evaluate the effects of kinase inhibitors, cells were pretreated with U0126, SB203580 or SP600125 for 45 ~ 60 min, and then exposed to NMDA with or without BL-M.

### 4.6. MTT Assay

The cell viability was measured using MTT reduction assay, as previously reported [14,32,33]. MTT was added to the treated cells at a final concentration of 1 mg/mL in phosphate-buffered saline (PBS) and the cells were incubated for 3 h at 37 °C. After removal of culture media from the wells, DMSO was added to dissolve the formazan crystal products. The absorbance was read at 550 nm using a microplate reader (SpectraMax M2^e^, Molecular Devices, Sunnyvale, CA, USA).

### 4.7. Measurement of Intracellular ROS

Spectrofluorometric determination of intracellular ROS levels was performed using DCFH-DA as a fluorescent probe [32,34]. In brief, cells were incubated with 10 μM DCFH-DA for 30 min at 37 °C. After removal of excess DCFH-DA, cells were washed with HCSS and then exposed to either Glu or NMDA for 2 h at 37 °C in the presence or absence of various concentrations of BL-M or memantine. Intracellular production of ROS was measured by the fluorescence detection of dichlorofluorescein (DCF) as the oxidized product of DCFH on a microplate reader (SpectraMax M2^e^, Molecular Devices) with an excitation wavelength of 490 nm and emission wavelength of 520 nm. The effects of BL-M and memantine on intracellular ROS generation were further evaluated using epifluorescence microscopy (Nikon, Tokyo, Japan) under the conditions described above. The fluorescence was detected using a FITC filter set and acquired images were processed with the help of Meta imaging system software (Molecular Devices).

### 4.8. Nuclear and Cytoplasmic Fractionation of Cultured Cells

Following the desired treatments, cultured cells were fractionated using the NE-PER™ Nuclear and Cytoplasmic Extraction Reagents Thermo Fisher Scientific (Waltham, MA, USA) following the manufacturer’s instructions. Nucleus and cytosol lysates were separated and stored at −80 °C until use.

### 4.9. Western Blotting

Following the desired treatments, the cells were washed with ice-cold PBS, pH 7.4, and lysed in a lysis buffer (10 mM Tris–HCl, pH 7.4; 150 mM NaCl; 2 mM ethylenediaminetetraacetic acid; 4.5 mM sodium pyrophosphate; 10 mM β-glycerophosphate; 1 mM NaF; 1 mM Na_3_VO_4_; 1% (*v*/*v*) Triton X-100; 0.5% (*v*/*v*) NP-40; and one tablet of protease inhibitor cocktail (Roche Diagnostic GmbH, Mannheim, Germany)) for 30 min on ice. Lysates were subjected to centrifugation at 14,000 rpm for 30 min at 4 °C, and the supernatants were isolated and stored at −80 °C until use. Equal amounts of protein (20 μg) were resolved by sodium dodecyl sulfate-polyacrylamide gel electrophoresis, and transferred to PVDF membrane (EMD Millipore, Billerica, MA, USA), as previously described [35,36]. The membranes were blocked for 2 h with 5% non-fat dried milk (BD Falcon, Sparks, MD, USA) in Tris-buffered saline containing 0.1% Tween 20, and incubated overnight at 4 °C with the specific primary antibodies in 5% bovine serum albumin (USB, Canton, OH, USA). Using the appropriate HRP-conjugated anti-IgG secondary antibodies, the immunoreactive bands were detected by a BioRad ChemiDoc XRS imaging system using Clarity^TM^ Western ECL Substrate (Bio-rad, Hercules, CA, USA).

### 4.10. Immunocytochemistry

Immunocytochemical staining of the cultured cells was performed according to protocol of Thermo Fisher Scientific [37]. In brief, following the desired treatments, cells were fixed with 4% paraformaldehyde for 15 min and permeabilized with 0.3% Triton X-100 for 5 min. Fixed cells were incubated with blocking solution (5% goat serum) for 2 h, followed by incubation with primary antibody in blocking solution. Next day, the solution was decanted and the cells were washed three times in PBS and incubated with the secondary antibody. Finally, cover glasses were removed from 24-well plate and mounted with ProLong Gold antifade reagent with DAPI Thermo Fisher Scientific (Waltham, MA, USA) on microscope slides (Marienfeld GmbH, Lauda-Königshofen, Germany), followed by staining with Alexa 488- and 555-conjugated secondary antibody Thermo Fisher Scientific (Waltham, MA, USA) and visualization with a Nikon confocal laser-scanning microscope.

### 4.11. Lipid Peroxidation Assay in Rat Brain Homogenates

Lipid peroxidation assay using rat brain homogenates as a lipid source was carried out as described [38,39]. Briefly, the reaction mixture containing rat brain homogenates, Fe^2+^, l-ascorbic acid and various concentrations of test compound was incubated at 37 °C for 1 h. Following addition of trichloroacetic acid (28% *w*/*v*) and thiobarbituric acid (1% *w*/*v*) to stop the reaction, the mixtures were heated for 15 min at 100 °C and centrifuged at 3000 rpm for 10 min at 4 °C. The absorbance of the transparent supernatant was measured at 532 nm using a SpectraMax M2^e^ microplate reader.

### 4.12. DPPH Radical Scavenging Assay

The assessment of the DPPH radical scavenging activity was measured as previously described [39]. In short, the reaction mixture containing various concentrations of the test compound and DPPH solution diluted in methanol was incubated at 37 °C for 30 min and the absorbance was measured at 520 nm.

### 4.13. Statistical Analysis

All experiments were repeated at least three times. Quantitative data were expressed as the mean ± S.E.M. The statistical analyses were performed using one-way ANOVA followed by Tukey’s test for all pairs *post hoc* test (Sigma Plot 12.5 software, San Jose, CA, USA). For all analyses, *p* < 0.05 was considered statistically significant.

## 5. Conclusions

In the present study, we demonstrated the neuroprotective effect of *N*-((3,4-dihydro-2H-benzo[h]chromen-2-yl)methyl)-4-methoxyaniline (BL-M), a newly synthesized chromene derivative, and compared to that of memantine, a non-competitive NMDA antagonist clinically used to treat AD. In addition, the mechanisms of action underlying the neuroprotective effect of BL-M were elucidated using primary cultured rat cortical cells. We found that BL-M inhibited excitotoxic neuronal cell damage induced by Glu or NMDA. Both BL-M and memantine exhibited comparable neuroprotection against NMDA-induced toxicity. However, their underlying action mechanisms were distinct. In contrast to memantine, BL-M exhibited marked antioxidant activity, which may contribute, at least in part, to its neuroprotective action. Furthermore, BL-M, not memantine, enhanced the ERK-mediated phosphorylation of CREB, which also plays a crucial role in mediating the neuroprotective activity. Based on our findings, BL-M may be beneficial for AD and other neurodegenerative disorders associated with excitotoxicity as well as oxidative stress.
